# Synthesis-Structure-Activity Relationships in Co_3_O_4_ Catalyzed CO Oxidation

**DOI:** 10.3389/fchem.2018.00185

**Published:** 2018-05-25

**Authors:** Kathleen Mingle, Jochen Lauterbach

**Affiliations:** Department of Chemical Engineering, University of South Carolina, Columbia, SC, United States

**Keywords:** CO oxidation, Colbalt oxide, combinatorial optimization, design of experiments (DOE), grain boundaries

## Abstract

In this work, a statistical design and analysis platform was used to develop cobalt oxide based oxidation catalysts prepared via one pot metal salt reduction. An emphasis was placed upon understanding the effects of synthesis conditions, such as heating regimen and Co^2+^ concentration on the metal salt reduction mechanism, the resultant nanomaterial properties (i.e., size, crystal structure, and crystal faceting), and the catalytic activity in CO oxidation. This was accomplished by carrying out XRD, TEM, and FTIR studies on synthesis intermediates and products. Additionally, high-throughput experimentation was employed to study the performance of Co_3_O_4_ oxidation catalysts over a wide range of reaction conditions using a 16-channel fixed bed reactor equipped with a parallel infrared imaging system. Specifically, Co_3_O_4_ nanomaterials of varying properties were evaluated for their performance as CO oxidation catalysts. Figure-of-merits including light-off temperatures and activation energies were measured and mapped back to the catalyst properties and synthesis conditions. Statistical analysis methods were used to elucidate significant property-activity relationships as well as the design rules relevant in the synthesis of active catalysts. It was found that the degree of grain boundary consolidation and anisotropic growth in fcc and hcp CoO intermediates significantly influenced the catalytic activity. By utilizing the discovered synthesis-structure-activity relationships, CO oxidation light off temperatures were decreased to <90°C.

## Introduction

Colloidal synthesis of nanoparticles is a versatile approach used to prepare materials with drastically different properties, potentially allowing them to be tailored for a wide range of applications in medicine, catalysis, and more (Qi, 2010). However, the difficulty in understanding relationships between synthesis conditions, desirable properties, and material performance is a limiting factor in the utility of these methods. An example of one such system are cobalt oxide nanoparticles. The properties of cobalt oxide nanomaterials, such as phase, morphology, and crystal faceting are known to affect properties such as active site density and redox properties which are important for catalyst reactivity in various oxidation and hydrogenation reactions, but the accessibility of these features through nanoparticle synthesis is poorly established (Jiang and Dai, 2010; Dangwal Pandey et al., 2012; Wang et al., 2012; Bai et al., 2013; Pang et al., 2013; Li and Shen, 2014; Iablokov et al., 2015; Ahn et al., 2016; Hu et al., 2016).

Cobalt and its oxides have a great deal of utility as precious metal replacements for numerous applications including CO and CO_2_ hydrogenation (Wang et al., 2011; Iablokov et al., 2012; Ahn et al., 2016), ammonia oxidation (Chinchen et al., 1987), NO_x_ traps, N_2_O decomposition, (Xue et al., 2007; Ma et al., 2013; Konsolakis, 2015) CO oxidation (Jansson et al., 2002; Yu et al., 2009; Wang et al., 2012; Lou et al., 2014) and other total hydrocarbon oxidations (Solsona et al., 2008; Liu et al., 2009; Salek et al., 2014). Of these reactions, CO oxidation is relatively simple and well understood- having been studied a great deal within the heterogeneous catalysis community. Presently, most researchers agree that CO oxidation on Co_3_O_4_ catalysts occurs via a Mars van Krevelen type mechanism where the active species is presumed to be Co^3+^ and less frequently, Co^2+^ (Mars and Van Krevelen, 1954; Wang et al., 2012). Because of this, CO oxidation is a useful probe reaction which can be used to glean structure sensitivities relevant to other reaction chemistries.

When considering the rate of a catalytic reaction, there are certain variables in its kinetic equation, such as the surface concentration of active sites (part of the pre-exponential factor) and the activation energy of the reaction, that are material and/or structurally specific (Landau et al., 2014). When a reaction is structure sensitive, these factors are subject to change when the catalyst size, shape, and morphology are tuned. For example, this could happen as a result of the exposure or occurrence of low coordination edge or defect sites.

The numerous shape, size, and structure sensitivities that have been reported for cobalt oxide nanocatalysts make them interesting materials to study. One such example is the ease at which Co_3_O_4_ can form electrophilic oxygen species (either O^−^ or ${\text{O}}_{2}^{-}$), as indicated by the concentration of these species on the oxide surface. Miyamoto et al. showed using rectangular NH_3_ pulse techniques that the surface oxygen contents were different for Co_3_O_4_ formed through the thermal decomposition of cobalt (II) carbonate when compared to Co_3_O_4_ formed through the hydrolysis of cobalt (II) nitrate (Miyamoto et al., 1984). This work was elaborated on by the Cao group who showed that cobalt oxides prepared using a soft reactive grinding (SRG) procedure had very high propane total oxidation rates which correlated with high concentrations of superficial electrophilic oxygen (O^−^) as evidenced by peak speciation in O_2_-TPD results (Liu et al., 2009). The same group suggested that enhanced stability and activity among certain samples could be further linked to increased surface defects created by long periods of grinding and evidenced by lattice distortion seen in XRD. Surface defects such as vacancies or steps and bulk extended defects such as dislocations, stacking faults, and twins are a reality of transition metal oxides which can potentially change the adsorption properties (e.g., surface oxygen bond strength) and reactivity of the surfaces which they inhabit (Sadykov et al., 2000). Work by Sadykov et al. definitively linked cobalt oxide defect density, which they defined indirectly by measuring the amount of weakly bonded oxygen, with catalytic activity for CO oxidation by demonstrating a linear dependence between the two when defect density was controlled via the preparation procedure (Sadykov et al., 2000).

The bulk oxygen mobility in cobalt oxide materials is another important property related to structure and catalytic reactivity (Chistoskova et al., 1999; Liu et al., 2009). Potentially, for metal oxides involved in catalytic reactions with mechanisms which utilize their surface redox functionality, this variable could drastically affect reaction rates (Royer and Duprez, 2011). This is due to an enhanced ability of the metal oxide to resupply the surface reaction with lattice oxygen. In recent years, researchers have begun to recognize the sensitivity of this variable to factors such as grain boundary consolidation, where the coalescence of grains can increase oxygen diffusion rates from the bulk, among other favorable effects (Landau et al., 2014).

The surface enrichment of the Co^3+^ cation in Co_3_O_4_ is perhaps the most heavily discussed cobalt oxide structure sensitivity in heterogeneous catalysis. Co_3_O_4_ has a regular spinel structure which contains a mix of octahedrally coordinated Co^3+^ cations and tetrahedrally coordinated Co^2+^ cations (Raveau and Seikh, 2012). The relative amounts of these cation types on the surface of Co_3_O_4_ nanoparticles can be tuned via the surface faceting of the nanoparticles and/or the degree of spinel inversion. Control over the Co_3_O_4_ cation distribution has been the subject of a great deal of research, as Co^3+^ surface enrichment and control over the Co^3+^/ Co^2+^ ratio have been established to dramatically affect specific reaction rates, stabilities, and light-off temperatures for many catalytic applications (Xie et al., 2009; Alvarez et al., 2012; Yu et al., 2013; Stelmachowski et al., 2014; Wen et al., 2014).

This structure sensitivity is, in one form or another, a consequence of the preparation conditions and variables used in the preparation of the cobalt oxide catalyst. However, most works dedicated toward the understanding of these relationships are extremely restricted in scope and don't consider the co-dependence of various synthesis variables and structural descriptors. Thus, only with careful experimental design where numerous variables of interest are simultaneously tracked is it possible to link preparation conditions, catalyst structure, and performance. To this end, factors known to be linked to cobalt oxide size and structure within the colloidal synthesis family were screened to establish their importance on structural properties and build predictive models with which to tune these properties (Wu et al., 2004; Wen et al., 2013). A series of 2-level factorial designs and response surface designs were used throughout the project with the aim of discerning the roles of factors involved in the synthesis of cobalt oxide nanoparticles through the thermal decomposition of cobalt acetate with diols in dibenzyl ether and discovering their influence on nanoparticle properties (size, morphology, phase) and catalytic performance in CO oxidation.

## Experimental

### Catalyst synthesis

A one pot colloidal synthesis technique was used to synthesize cobalt oxide nanoparticles with varied shape, size, and phase. Specifically, cobalt acetate was thermally decomposed in a high boiling point solvent (dibenzyl ether) and reduced with a polyol (1,2-dodecanediol). Both polyvinylpyrrolidone (PVP) and oleic acid (OA) were investigated as capping agents to modulate nanoparticle growth by preventing excessive Oswald ripening. The relative concentrations of these reagents together with the temperature ramp and aging temperature of the reaction mixture comprised the parameters investigated using statistical design of experiments. A thermocouple submerged in the nanoparticle solution was used as an input to a PID temperature control system interfacing with a heating mantle. Thermal aging was carried out in a reflux apparatus under a constant purge of N_2_ gas. After the reaction mixture cooled to room temperature the nanoparticles were precipitated using repetitive centrifugation and washed with acetone three time each. Prior to characterization and catalytic testing, all samples were dried at 110°C and calcined at 550°C for 14 h in air to ensure complete oxidation to the spinel structure, Co_3_O_4_.

### X-ray diffraction

X-Ray Diffraction was carried out for all samples, both before and after calcination, in a Rigaku Miniflex II equipped with a Cu-Kα X-ray source and a high-speed silicon strip detector. Scans were completed between 10 and 90° 2θ angle at a rate of 2°/min with step size of 0.02°. Analysis of XRD patterns for all samples was carried out to verify the existence of a crystalline phase and identify the crystal structure and grain size of the phase. In cases where the relative intensity of XRD peaks could be used to indicate preferential growth or crystal faceting, this variable was tracked as well. In other cases, where more than one phase was present, an analysis of the volume fraction of each phase was carried out by utilizing the relationship of direct proportionality between XRD intensity and phase concentration (Norrish and Taylor, 1962).

### Electron microscopy

Transmission electron microscopy (TEM) was carried out to ascertain the morphology and particle sizes of the cobalt oxide nanoparticles before and after calcination. A Hitachi H-8000 TEM operated at 200 kV was used to take standard micrographs and an aberration corrected JEM2100F-200kV FEG-STEM/TEM was used to achieve atomic resolution images of selected samples.

### Catalytic activity tests in high-throughput reactor

Tests of catalyst reactivity were carried out in a 16-channel high throughput reactor with a parallel Fourier transform infrared spectroscopy (FTIR) imaging system, which is described in detail elsewhere (Snively et al., 1999, 2000, 2001a,b, 2002; Hendershot et al., 2005; Sasmaz et al., 2015). CO oxidation activity tests for the Co_3_O_4_ nanoparticles were completed using 50 mg of catalyst per channel under a 2% CO/8% O_2_/N_2_ gas stream at a space velocity of 60,000 mL·^−1^· ${\text{g}}_{\text{cat}}^{-1}$. Each channel had flow rates within 6% of the flowrate setpoint measured at the reactor outlets post FTIR analysis and identical inlet gas compositions. The temperatures of each catalyst bed, measured individually, could be tuned to equivalent set points ±1.6°C. Typically, the experiments included the measurement of CO conversion at 25°C intervals using FTIR images of the effluent gas stream and gas phase calibrations for the CO stretch at 2,150 cm^−1^ and the CO_2_ asymmetric stretch at 2,350 cm^−1^. Calibration R^2^ values were 0.99 or above for all 16 channels and the carbon mass balance error was closed with an average error of 0.5% over all reaction and validation data. Light off temperatures (the temperature required to achieve 50% CO conversion-herein referred to as T50) and reaction rate data were collected for 50 unique samples and replicates totaling more than 100 catalysts. No pretreatment was carried out onstream prior to catalytic testing, but samples were ensured to be fully oxidized by the ex-situ calcination, as described previously. No deactivation was observed for the duration of catalyst testing, which typically lasted around 5 h.

## Results and discussion

### Catalyst synthesis

Colloidal synthesis factors studied were selected based on experience and literature review. They included the concentrations of a cobalt acetate metal precursor, the type of protecting agent (PVP or oleic acid), and a diol reducing agent, as well as the synthesis temperature and heating ramp employed (Wu et al., 2004; Wen et al., 2013). A description of the variables and levels explored via a 6-factor, 2-level factorial design and a 2-factor central composite response surface design (CCD) are outlined in Tables 1, 2, respectively. Standard methodology for experimental design, term selection, model development, and Analysis of Variance Analysis (ANOVA) were followed (Montgomery, 1991; Khuri and Cornell, 1996). It should be noted that in the factorial design outlined in Table 1, center point runs were also completed. Prior to catalytic testing, all samples were dried at 110°C and calcined at 550°C for 14 h in air to ensure complete oxidation to the spinel structure, Co_3_O_4_.

**Table 1:** Initial factorial screening design.

**Parameter**	**Low Level (–)**	**High Level (+)**
A: Aging temperature (°C)	240	270
B: Heating rate (°C/min)	1	10
C: Surfactant (M)	0.05	0.25
D: Cobalt acetate (M)	0.05	0.15
E: Reductant (M)/Cobalt(M)	2	8
F: Surfactant Type	OA	PVP

**Table 2:** Follow-up response surface design (CCD).

**Parameter**	**Low Level (–)**	**High Level (+)**
A: Aging temperature (°C)	200	300
B: Cobalt Acetate (M)	0.02	0.1

### Control of structure and morphology

X-ray diffraction and TEM studies were carried out to resolve the influence of the synthesis factors on the bulk crystal structure, morphology, grain size, and particle size of the catalysts prior to calcination. Crystalline phase was determined for each sample based on the relationship between lattice parameters, miller indices, and d-spacing for cobalt, and Scherrer's equation was applied to approximate crystallite size from peak broadening (Azaroff and Buerger, 1958; Henry et al., 1960). Size distribution and morphology was studied using TEM as well as STEM for selected samples. It was found that CoO particles in the 1–100 nm size range were synthesized with both cubic and hexagonal close packed crystal structure (herein referred to as f-CoO and h-CoO, respectively). Additional phases observed in the factorial design were alkoxide type and layered-hydroxy structure type precursors.

**Figure 1:**
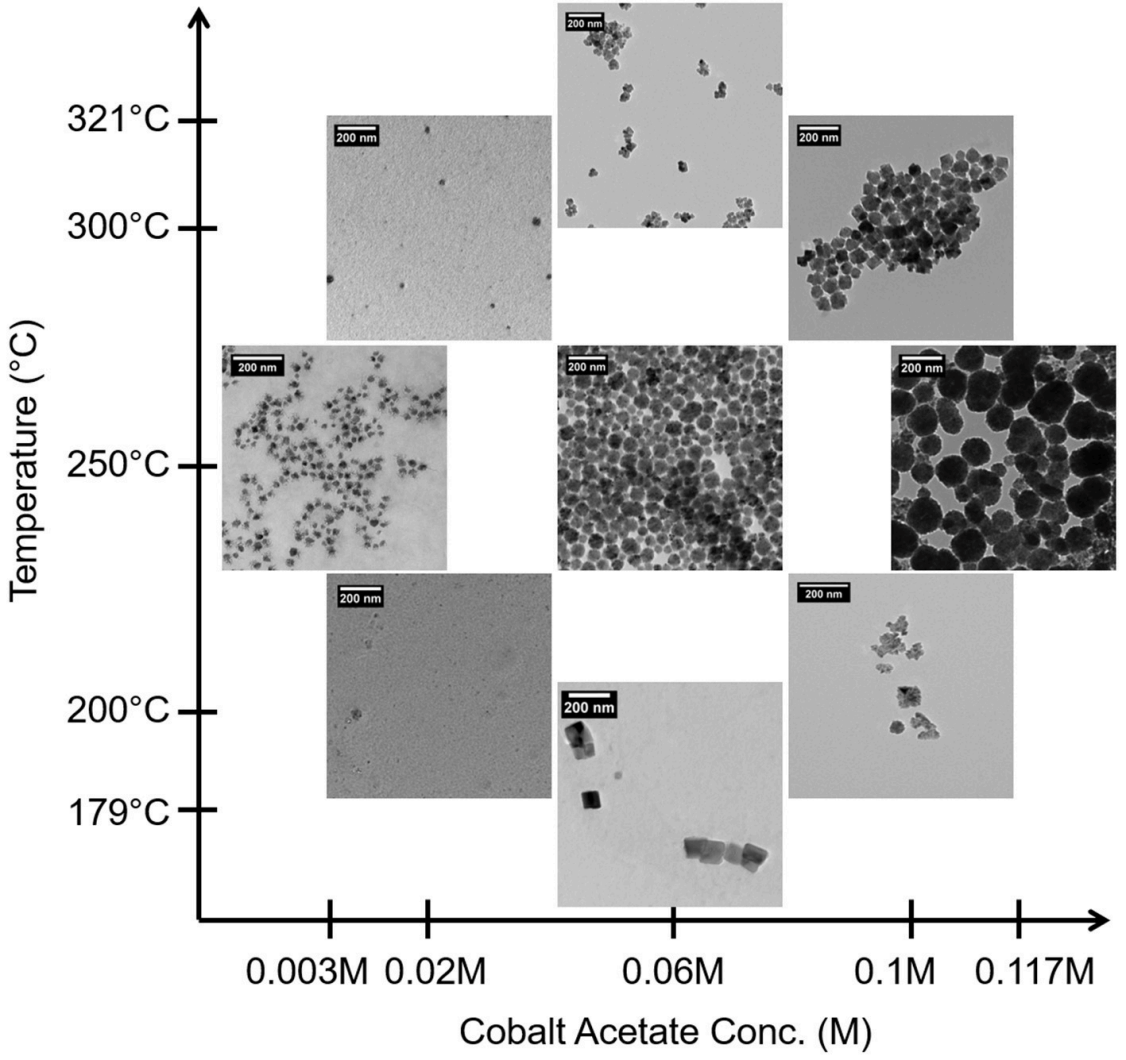
TEM images of CCD CoOx samples.

Figure 1 contains TEM micrographs of selected samples synthesized according to the levels selected for the CCD (Table 2) and illustrates the variability in the size and morphology of particles obtained as a function of just cobalt acetate concentration and aging temperature. A clear correlation where increasing cobalt acetate concentration increases particle size and polydispersity is visually evident, but a more refined understanding of how metal concentration and aging temperature affect particle sizes and dispersions requires statistical analysis. By carrying out a least-squares regression and pareto analysis of our design variables and corresponding terms, we can learn how much the population mean of particle size and dispersion changes with respect to each term when scaled by standard error. In doing this, we can see which terms have the greatest effect on the particle size and dispersion when error is accounted for and be confident that these terms are important when control over particle size and dispersion are desired. This analysis confirms that cobalt acetate concentration is the most important factor studied in the control of particle size and dispersion. However, it also suggests that the effect of increasing cobalt acetate concentration is sensitive to the aging temperature employed. At aging temperatures below 250°C, increasing cobalt acetate concentration has the effect of changing the crystal structure from f-CoO to h-CoO and layered cobalt hydroxy and alkoxy acetates whilst at 250°C and above, increasing cobalt acetate concentration increases size and polydispersity of the particles while maintaining a f-CoO phase.

**Figure 2:**
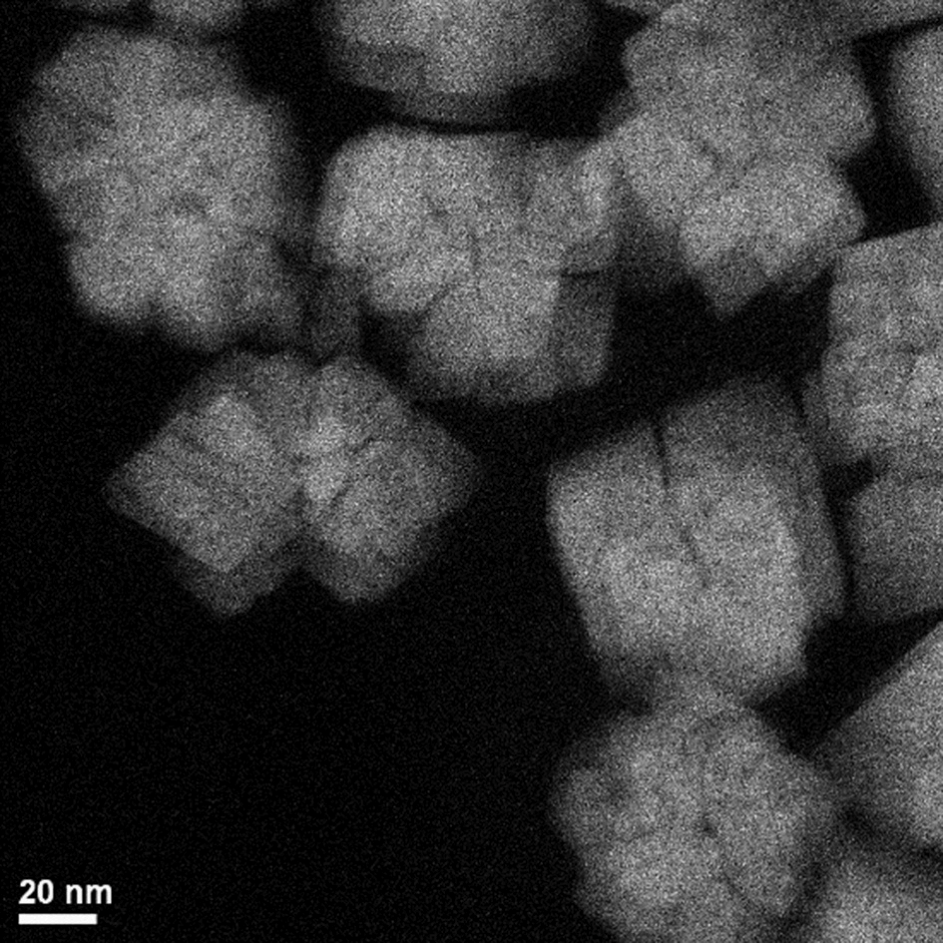
TEM image showing grain boundary consolidation in f-CoO nanoparticles.

XRD analysis aided in elucidation of the phases formed and the grain sizes existing within the particles. Interestingly, the CoOx grain sizes measured were not found to change in the same ways as the particle sizes measured with TEM. In the case of grain size, the aging temperature, cobalt acetate concentration and surfactant concentration were found to be important, and the largest grain sizes were formed at low concentrations of cobalt acetate and low aging temperatures or high concentrations of cobalt acetate and high aging temperatures. Thus, CoO particles with increasingly small grain sizes were obtained at intermediate aging temperatures and cobalt salt concentrations. Incidentally, the same region of the design space led to the fabrication of large particles up to 80 nm in size. From this information, together with visual inspection of TEM images such as those in Figure 1, it is clear that a certain balance of cobalt salt concentration and aging temperature, as well as sensitivity to other variables studied in the screening design (Table 1) can lead to grain boundary consolidation for f-CoO structures. Consolidation was not observed for the h-CoO or LHS structures synthesized at lower temperatures. An example of this type of morphology is shown in Figure 2, where small particles are coalesced into stable aggregates in the case of f-CoO nanoparticles synthesized at 250°C with 0.06M cobalt acetate. Intuitively, we might expect this to happen when the f-CoO growth rate exceeds the nucleation rate. The highest degrees of grain boundary consolidation were found to exist close to the center point of the central composite design (Table 2) at 0.06M cobalt acetate and a 250°C aging temperature. Moving away from this region in any direction was found to decrease the level of grain consolidation.

Numerous schemes have been proposed to quantify the grain boundary coalescence in nanomaterials which use a combination of experimentally measured values of BET surface area and the diameter of grains and/or particle aggregates measured with TEM and XRD (Landau et al., 2014). Henceforth, the scheme proposed by Tsybulya et al (Tsybulya, 1995) will be used to define grain boundary consolidation as the ratio between the particle size, including particle aggregates, measured with TEM and the grain size measured by applying Scherrer's equation to the X-Ray Diffraction (XRD) pattern (Azaroff and Buerger, 1958; Henry et al., 1960). This relationship is shown in Equation (1).

(1) $$\eta =\frac{D_{TEM}}{D_{XRD}}$$

Based on the phases fabricated in the totality of experiments (including both screening design and CCD), relationships between synthesis factors and crystalline phase can be elucidated. Specifically, for the factorial screening design (Table 1), aging temperatures were between 240°C and 270°C, all samples were comprised of either h-CoO, f-CoO, or a mix of the two. In this region of the design space, it was found that the %h-CoO in the sample was increased by increasing the aging temperature from 240 to 270°C, provided that surfactant concentration and cobalt salt concentrations were low (on the order of 0.05M). Additionally, it was found that increasing the concentration of 1,2-dodecanediol in the synthesis decreased the %h-CoO. The finding that high temperature could induce a kinetically controlled regime leading to h-CoO crystals which would be diminished by increasing the level of surfactant in the synthesis, effectively slowing the thermal decomposition rate, is comparable to the results of others. For example, Nam et al found that the thermal decomposition of cobalt acetylacetonate at rapid ramp rates and high temperatures led to h-CoO while low ramp rates, low temperatures, and long aging times led to c-CoO (Nam et al., 2010). Additionally, they found that the addition of dichlorobenzene to the reaction mixture had the effect of first increasing the aspect ratio of h-CoO to yield rods and finally yielding c-CoO at higher concentrations. In contrast, no dependence was found in this work on heating rate or aging time.

**Figure 3:**
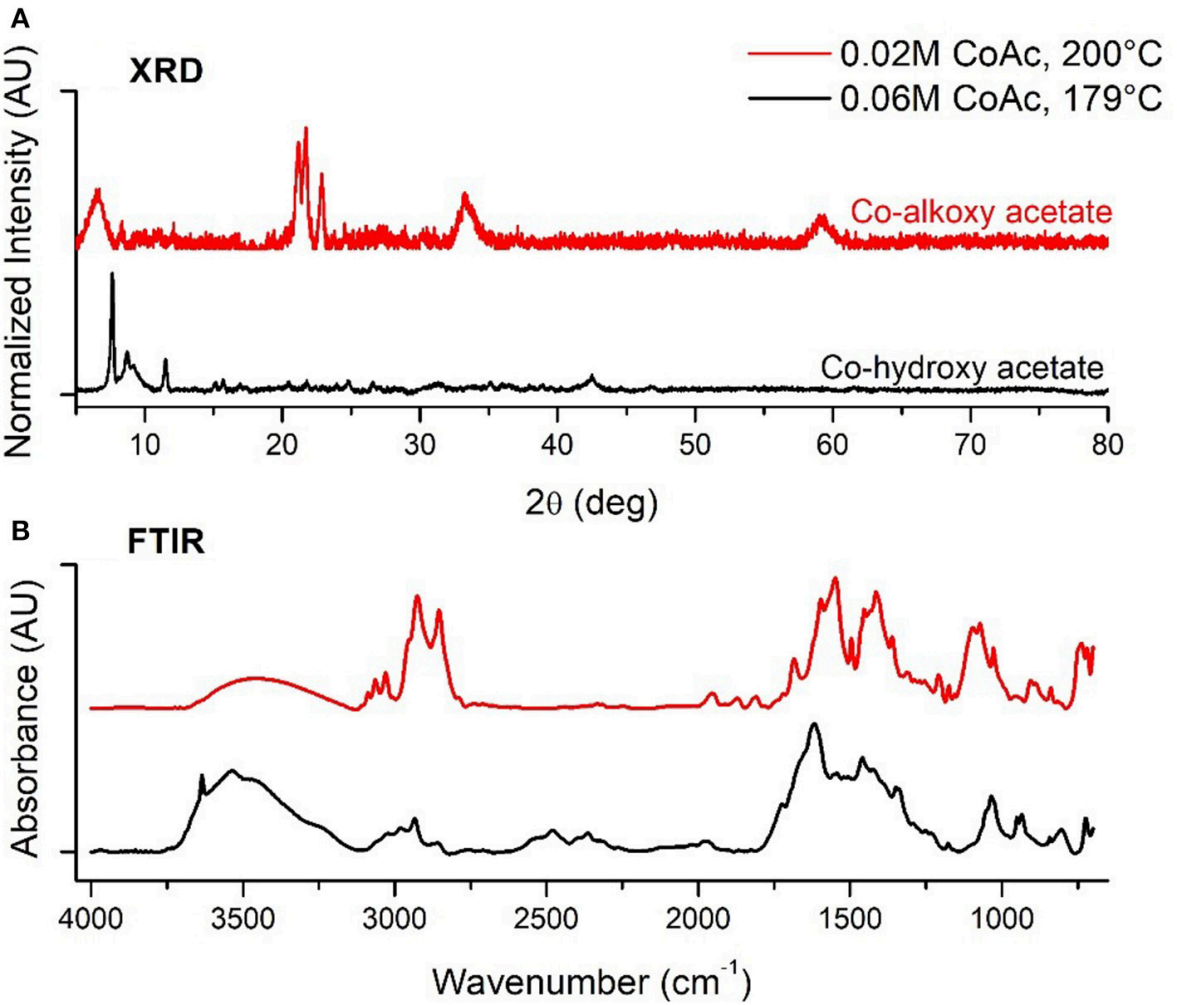
**(A)** XRD and **(B)** FTIR of Co alkoxy and hydxroxy acetate structures formed at low temperature.

Samples synthesized at CCD levels (Table 2), over a 179–321°C temperature range, led to four distinct outcomes in terms of crystalline phase. Intermediate cobalt acetate concentration and low temperature (179°C and 0.06M) yielded a mixture of large cubes and rods with edge lengths and diameters of 93.6 ± 10.6 nm which was indexed to match a layered cobalt hydroxy structure [Co(OH)(CH_3_COO), PDF#22-0582] (Doremieux, 1967). Similar morphologies were prepared by Poul et al in their studies of layered hydroxide metal acetates which they describe as being structurally similar to hydrozincite structures layered with unidentate coordinated acetate ions (Poul et al., 2000). This type of structure is comparable with our material as evidenced by XRD and *ex-situ* FTIR spectral analysis shown with black in Figures 3A,B, respectively, which confirms the existence of blue shifted acetate ν_as_ (C=O) and ν_s_ (C=O) bands indicative of acetates with unidentate coordination to Co^2+^ (Chakroune et al., 2005). At low aging temperature and cobalt acetate concentration (200°C and 0.02M) spherical particles with a TEM measured diameter of 10.3 ± 2.6 nm were synthesized. Repeat syntheses under these conditions led to materials that were either x-ray amorphous or structurally similar to cobalt alkoxide and alkoxyacetate phases seen by others characterized by the intense low angle diffraction peaks of layered structures (Poul et al., 2000; Chakroune et al., 2005). The XRD reflections of this phase are shown in red in Figure 3A. In this type of lamellar structure, cobalt-oxygen units are separated by alcoholate anions originating from the polyol solvent or reducing agent as well as the acetate anion, in some cases. At the 200°C and 0.02M CoAc synthesis condition, splitting of the ν_s_ (CH) and ν_as_ (CH) bands originating from the diolate anion in ex-situ FTIR, shown in red in Figure 3B, suggests that the Co^2+^ is chelated by the diol as well as being coordinated in a unidentate manner to the acetate anion (Chakroune et al., 2005). At low temperature and higher cobalt acetate concentration (200°C and 0.1M), particles of 57.2 ± 20.2 nm were formed with mixed h/f-CoO structures (PDF#s 89-2803 and 78-0431). At temperatures of 240°C and above over the entire range of cobalt acetate concentrations investigated only f-CoO crystals were obtained with particle sizes ranging from 22.2 to 82.7 nm.

The fabrication of metastable phases is consistent with a solution phase reduction of cobalt salt which exists in a kinetic rather than thermodynamically controlled regime (Song et al., 2006; Lu et al., 2014). Additionally, aging temperatures at 200°C or below are likely not high enough to induce thermal decomposition of the CoO precursor meaning that the final product obtained, a layered hydroxy acetate may be considered an intermediate in the formation of CoO. For these reasons, it is not completely surprising that cobalt based nanostructures of a number of different phases were obtained over the entirety of the designs; including h-CoO, f-CoO, h/f-CoO mixtures, cobalt hydroxy acetate, and cobalt alkoxy acetate based structures.

**Figure 4:**
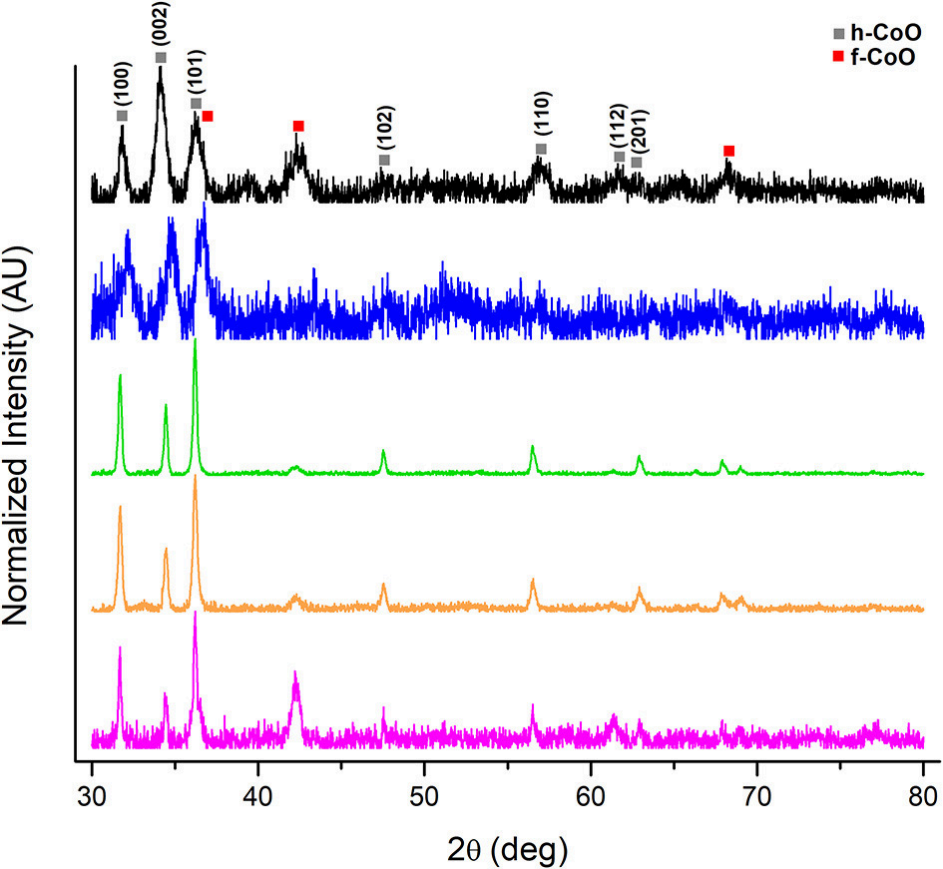
XRD patterns of h-CoO nanoparticles prepared at varied synthesis conditions showing preferential growth along the c-axis (black: 200°C, 5.5°C/min, 0.1M CoAc, blue: 270°C, 1°C/min, 0.15M CoAc, green: 270°C, 10°C/min, 0.05M CoAc, orange: 270°C, 10°C/min, 0.15M CoAc, pink: 270°C, 1°C/min, 0.05M CoAc).

Interestingly, the collection of h-CoO and h-CoO/f-CoO structures originating from both the screening and central composite design were found to differ in their crystal faceting evidenced by XRD. The ratio between h-CoO (002) and (100) XRD reflections varies between samples, which is suggestive of preferential growth along the c-axis of the wurtzite structure (Tian et al., 2003; An et al., 2006). An example of h-CoO nanoparticles with varying degrees of faceting are shown in Figure 4 where the bottom (pink) XRD pattern is representative of no faceting and the top (black) pattern represents the highest observed degree of faceting. All samples shown in Figure 4 were prepared at aging temperatures between 200 and 270°C, where the degree of faceting is primarily changing as a function of the heating rate (1–10°C/min) and cobalt acetate concentration (0.05–0.15M). Since f-CoO was often in coexistence with h-CoO, the volume of each phase was accounted for when considering the effect of this variable on activity. Interestingly, the f-CoO/h-CoO ratio alone was not found to have a correlative relationship with CO oxidation activity. This is likely because changes in this variable were convoluted with changes in f-CoO grain boundary consolidation and h-CoO faceting, both of which more strongly influenced the catalytic properties.

The degree of h-CoO faceting [measured via the (002)/(100) XRD intensity ratio] was found, from statistical analysis, to be very sensitive to aging temperature, heating rate, and cobalt salt concentration. Specifically, considering an aging temperature of 270°C, increased growth along the c-axis could be achieved by decreasing the heating rate whilst at high (>0.1 M) cobalt acetate levels or increasing the cobalt acetate concentration whilst using low (< 5°C/min) heating rates. Additionally, increasing the aging temperature when low heating rates (1°C/min) were employed decreased growth in the c-direction while increasing aging temperature when high heating rates (10°C/min) were employed increased the degree of faceting. In order to maximize the anisotropic growth in h-CoO crystals, a combination of low aging temperature (< 250°C) and low heating rates (< 6°C/min) were most effective in forming h-CoO in favor of c-CoO, and under these conditions, increasing the cobalt acetate concentration led to more faceted particles. While c-axis growth is an intrinsic property of wurtzite crystal systems it is diminished when the process is not kinetically driven. Thus, establishing faster growth kinetics through temperature, heating regimen, and increased metal salt concentration has generally been found to produce high surface area, more anisotropic wurtzite crystals (Peng et al., 2000; Pacholski et al., 2002; Nam et al., 2010).

**Figure 5:**
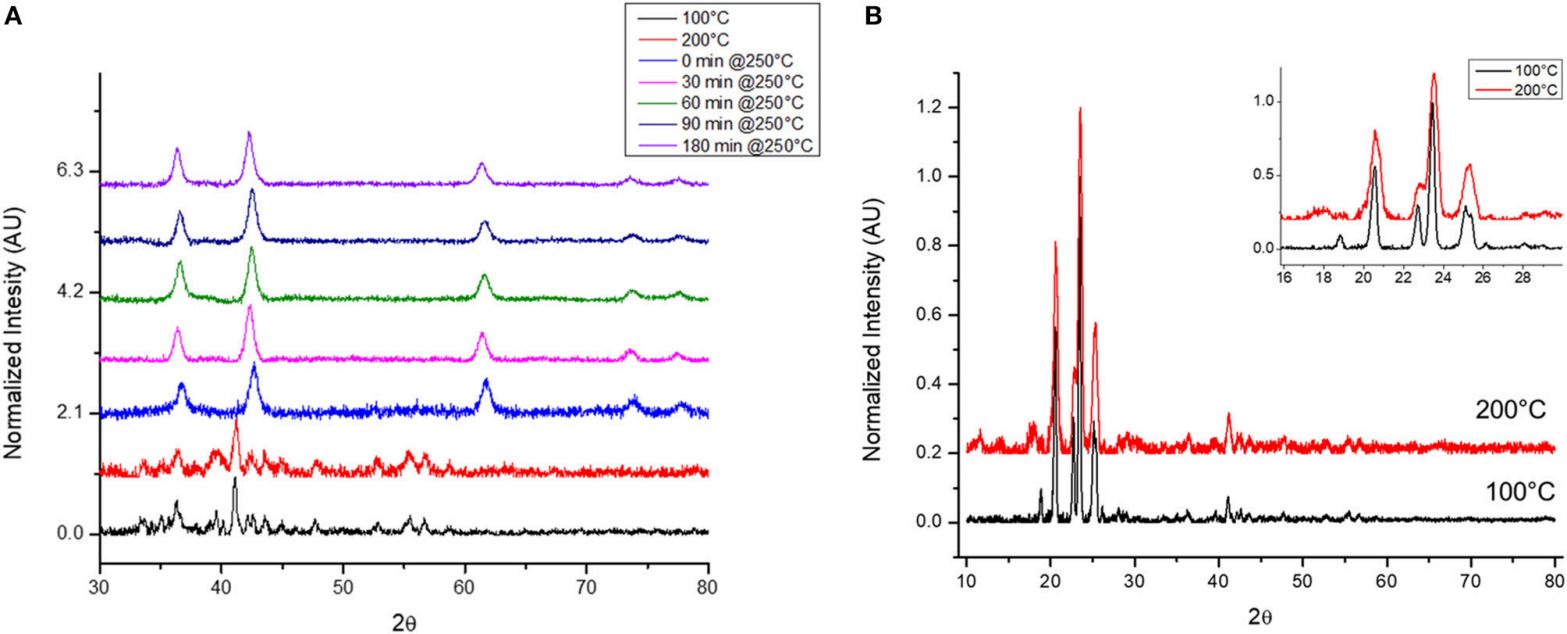
**(A)** Time-resolved synthesis at 0.06M CoAc, 250°C for f-CoO nanoparticles and **(B)** low-angle reflections.

To understand the formation of h-CoO it is necessary to consider how this phase is related to other phases fabricated. Some work has suggested that f-CoO could be an intermediate in the oxidation of h-CoO to Co_3_O_4_ (Nam et al., 2010), which would help explain the common co-existence of these phases in this work. Another possibility is that these phases arise from distinct synthesis intermediates arising when cobalt acetate partially or fully decomposes in solution and complexes with the diol reducing agent, producing the same or similar intermediate coordinated CoO structures such as those which form at low temperatures as previously discussed. Time resolved synthesis experiments were carried out to address these possibilities focused on the center point of the CCD (0.06M CoAc, 250°C) which had the terminal phase of f-CoO. Aliquots were sampled from the reflux vessel during the heat-up phase at 100°C, 200°C, and every 30 minutes once the reaction reached the 250°C aging temperature. The XRD patterns of these aliquots are shown in Figure 5A. Prior to reaching 250°C a unique diffraction pattern was observed with low index peaks between 20 and 30°2θ comparable to, but not identical to, the layered alkoxy-acetate structures previously discussed. The low angle XRD reflections of the sample aliquots collected at 100 and 200°C can be seen in Figure 5B. Once aging at 250°C commenced, f-CoO was formed immediately, and no further phase change was observed for the duration of refluxing which totaled 180 minutes, providing no evidence that f-CoO formation proceeds through a h-CoO intermediate. Additionally, analysis of the XRD peak broadening with Scherrer's equation and TEM image analysis showed that grain size and particle size remained virtually unchanged for the duration of the 180 minute thermal aging.

### Effect of surfactant choice

Oleic acid (OA) is a long chain fatty acid with the formula C_18_H_34_O_2_ and is commonly employed as a capping agent for hydrophobic nanoparticles. It forms a strongly interacting layer around the particles which acts a barrier to mass transfer and limits magnetic and van der Waals interactions (Wu et al., 2004; Repko et al., 2011; Jovanovic et al., 2014). In comparable solution phase synthesis reports of cobalt based nanoparticles, adding 0.25M OA was found to decrease the average particle size from 18 to 6 nm (Jovanovic et al., 2014). In the same study, FTIR experiments led to the conclusion that stabilization was achieved via bridging bidentate interactions between OA and Co metal atoms. Polyvinylpyrrolidone (PVP) is a high molecular weight (Mn of approximately 40,000 Da) non-ionic polymer with C=O, C-N, and CH_2_ functionality. Since PVP has mixed functionality, having both a highly polar, hydrophilic amine group on the pyrrolidone ring and a hydrophobic alkyl group, it is a suitable protective agent for many different nanoparticle and solvent systems (Koczkur et al., 2015). Statistically, there was no effect of using PVP vs. OA as a protective agent in terms of particle size, grain size, crystalline phase, or catalytic activity of the prepared materials. This suggests that the mechanism of protection for both PVP and OA originates from the hydrophobic carbon chains interacting with one another to create steric hindrances.

### CO oxidation activity

**Figure 6:**
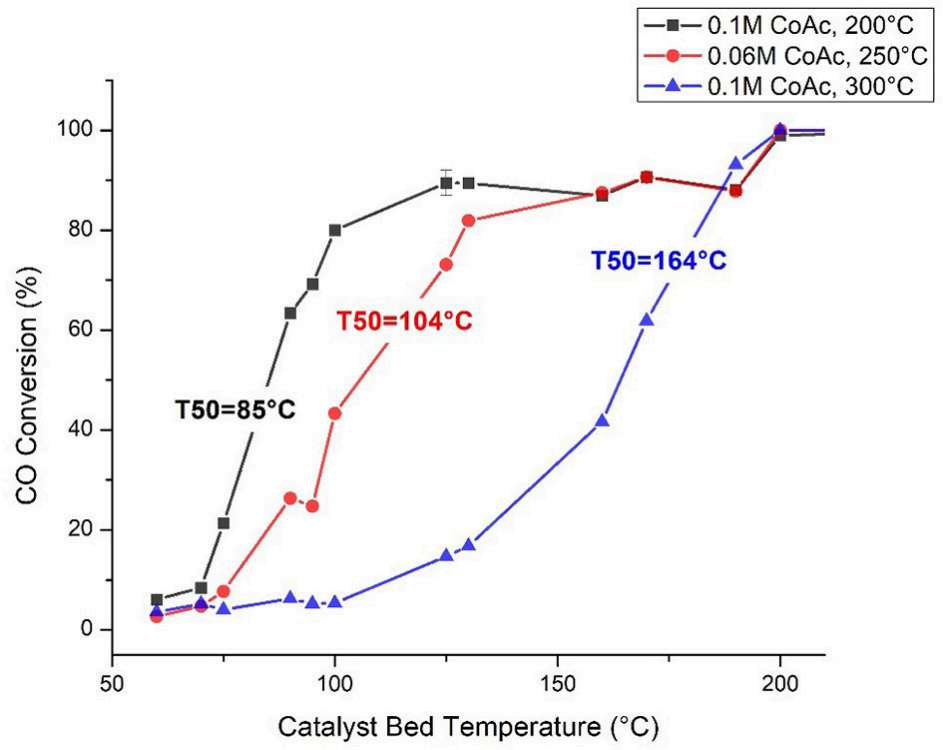
CO oxidation light-off curves for selected catalysts.

The highest CO oxidation activities, coinciding with the lowest light off temperatures were achieved with catalysts synthesized at higher cobalt acetate concentrations and intermediate synthesis temperatures. This conclusion is supported by the statistical analysis of the activity data via the factorial screening and response surface designs (Tables 1, 2) which show that cobalt acetate concentration and its interaction with synthesis temperature were the most important variables. Additionally, by measuring apparent activation energies using the high-throughput reactor (Table 3), it was observed that the activation barrier for CO oxidation was also changing as a function of synthesis conditions. The simultaneous change of activity and apparent activation energy indicates that the increased activity is not simply due to a higher fraction of exposed catalytically active centers, but that the active center is different. Figure 6 shows three selected catalysts with varied light off temperatures and activation energies. More specifically, one without any grain boundary consolidation or h-CoO faceting, one with an intermediate degree of grain boundary consolidation (η = 6) and one with the highest measured degree of h-CoO faceting measured with XRD.

**Table 3:** Synthesis conditions and structural properties of selected catalysts.

**Synthesis condition**	**Phase (pre-calcination)**	**η**	**T50(^°^C)**	**Ea (kJ/mol)**
0.1M CoAc, 200°C	h-CoO	1	84.3 ± 1.6	30.2 ± 5.0
0.06M CoAc, 250°C	c-CoO	6	105.2 ± 1.5	50.8 ± 4.4
0.1M CoAc, 300°C	c-CoO	1	162.3 ± 3.0	67.4 ± 5.8

### Synthesis-structure-activity relationships

**Figure 7:**
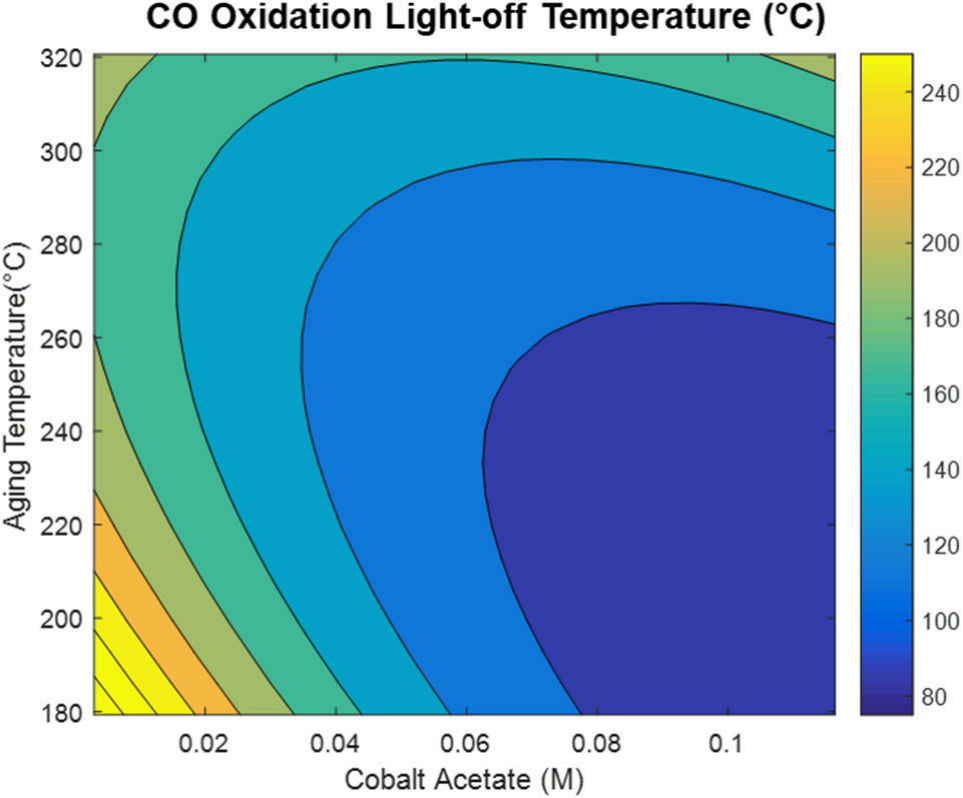
Light-off temperature contour map developed with CCD.

**Figure 8:**
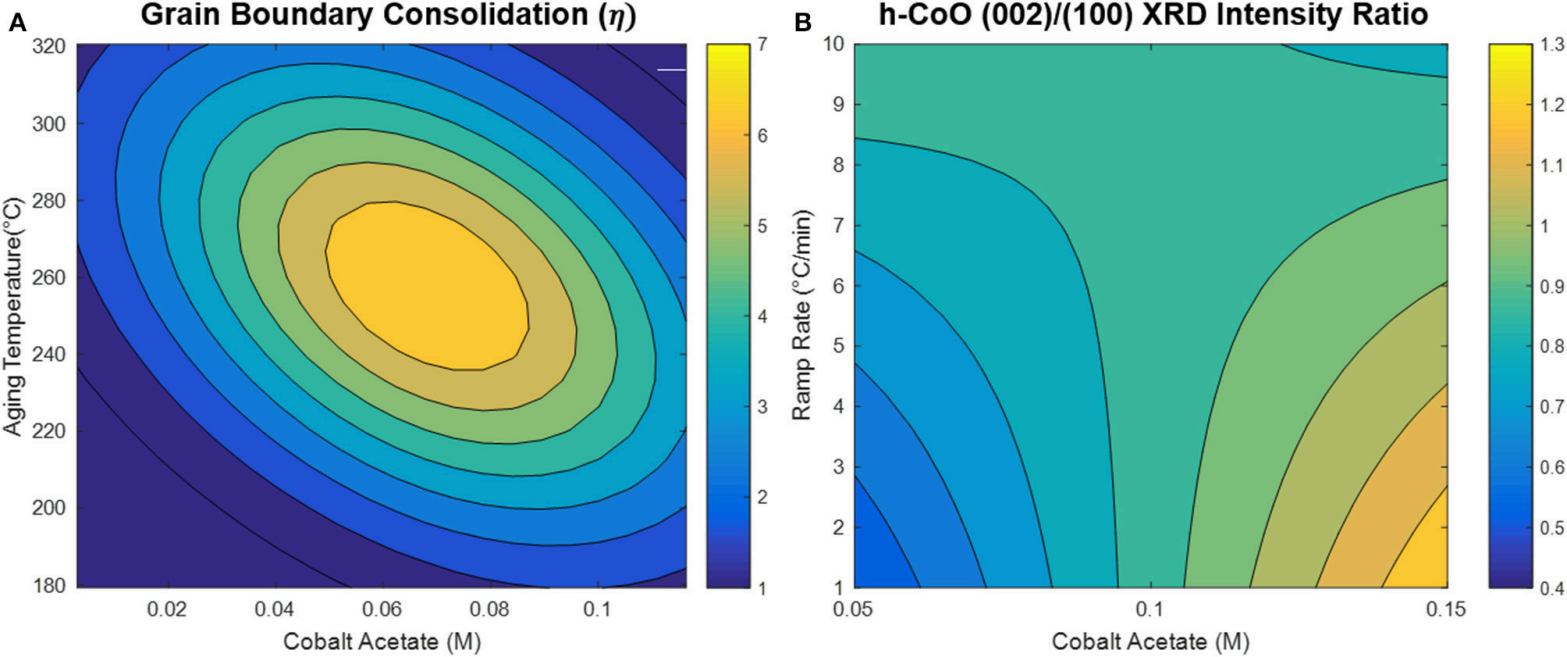
**(A)** Grain boundary consolidation contour map developed with CCD and **(B)** h-CoO (002)/(100) XRD intensity ratio contour map generated with screening design.

The model generated via the central composite design around cobalt acetate concentration and aging temperature shows that the CO oxidation light-off temperature appears to be decreased by increasing the cobalt acetate concentration and decreasing the aging temperature during the synthesis, as depicted in Figure 7. Comparison of the dependence of light-off temperature on synthesis variables to the dependence of structural properties points to possible explanations for observed variations in catalytic activity. An interesting example is the variation of grain boundary consolidation (η) on synthesis variables, as depicted in Figure 8A. As previously stated, η is maximized at the center point of the CCD (0.06M CoAc, 250°C), and follows similar curvature to that of the light-off temperature contour in Figure 7. One notable difference is that the optima in the two contour plots do not exactly coincide. This can be explained by the optimum light-off temperature being associated with a faceted h-CoO intermediate having been formed at 0.1 M and 200°C. In fact, cross-checking this result with the formation of other faceted h-CoO nanoparticles in the screening design confirms that these types of structures tend to be formed at low ramp rates, low aging temperatures and high CoAc concentrations. This is depicted in Figure 8B, where optimum growth along the h-CoO c-axixs coincides with low ramp rates and high CoAc concentration.

**Figure 9:**
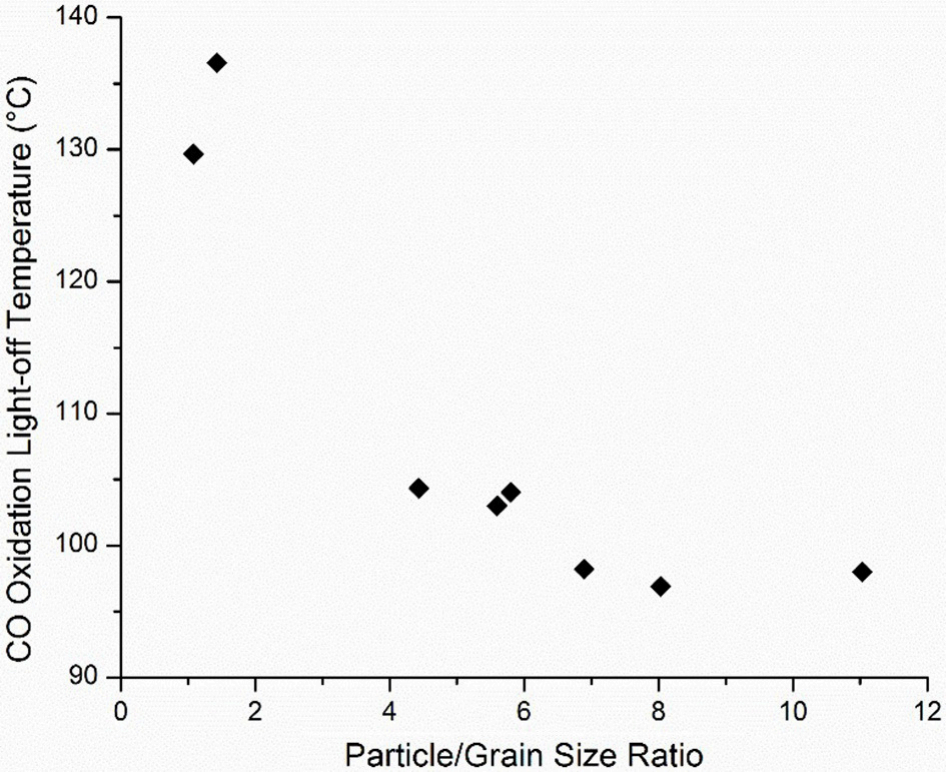
Relationship between CO oxidation activity and η.

To further investigate these correlations, the CO oxidation light-off temperature was plotted against η for all samples which exhibited this characteristic. It should be noted that this relationship was identified during the analysis of the response surface design, but to further validate the relationship, catalysts from the entirety of the experimental design sets were sampled. As shown in Figure 9, as η increases, CO oxidation light off temperature decreases, although diminishing effects are observed at η > 8. It should be noted that in this analysis η is changing mainly as a function of grain size and particle size and that other structural variables, such as the pre-calcination phase, are constant. Moreover, neither particle size nor grain size independently showed a significant correlation with catalytic activity, which is in contrast to other reports that have suggested that smaller particles favor activity due to their greater number of low coordination atoms (Dangwal Pandey et al., 2012; Mankidy et al., 2014). Here, by directly comparing those two effects, it is evident that the consolidation of grain boundaries is significantly more important than the particle size alone. High activities in oxidation reactions have been previously correlated with metal oxide grain boundaries with the primary reason being fast oxygen diffusion from bulk to surface to participate in the surface reaction with close proximity active sites. Similar findings have also been reported for other metal oxides (Sadykov et al., 2000; Wolf, 2001; Royer et al., 2005; Royer and Duprez, 2011).

**Figure 10:**
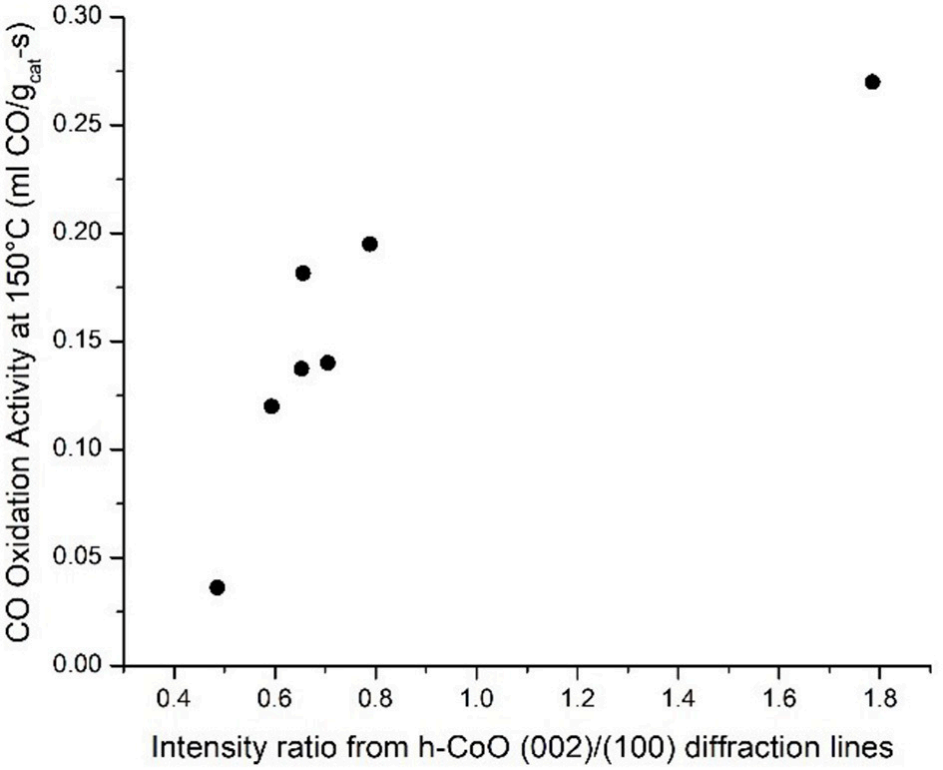
Relationship between CO oxidation activity and h-CoO faceting.

**Figure 11:**
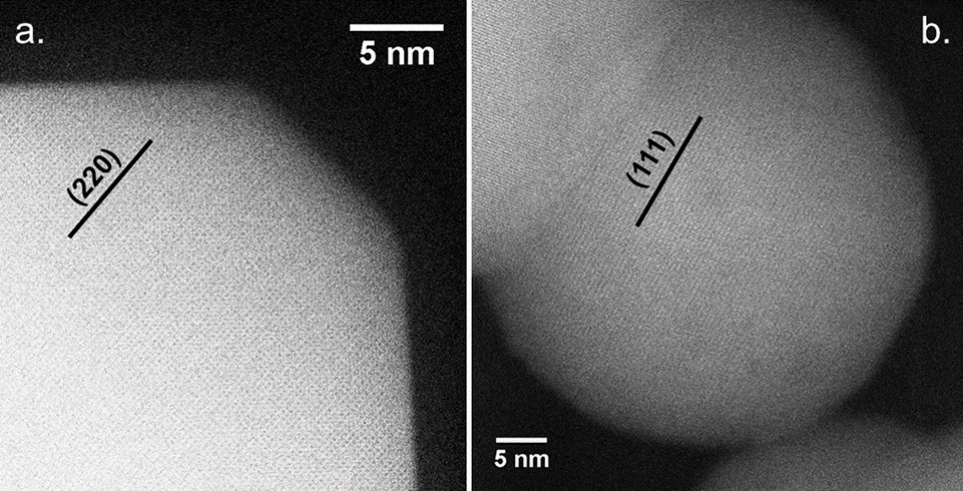
HRTEM images of Co_3_O_4_ originating from **(a)** faceted h-CoO and **(b)** f-CoO.

A similar assessment can be made regarding the coincidence of high CO oxidation activity with the formation of faceted CoO hcp particles by considering all relevant samples. In fact, plotting this variable with respect to the CO oxidation rate at 150°C confirms the relationship between higher h-CoO XRD intensity ratios and CO oxidation activity, as shown in Figure 10. Since the CoO catalysts are calcined before being tested for CO oxidation, it is of interest to understand how a highly faceted h-CoO nanoparticle evolves into a more active Co_3_O_4_ catalyst than its h-CoO counterpart with a low degree of faceting. Intuitively, one might reason that higher CO oxidation rates arising from faceted h-CoO structures must be related to a greater number of active sites being preferentially exposed on the Co_3_O_4_ structure after calcination, especially when the expectation is that the morphology of the CoO intermediate would be preserved (Nam et al., 2010). Indeed, HRTEM images of the highly faceted h-CoO after calcination to Co_3_O_4_ reveal surface termination by (110) facets rather than the (111) and (001) facets typical of Co_3_O_4_ nanoparticles. This is evidenced in Figure 11a which shows a faceted Co_3_O_4_ sample originating from faceted h-CoO, prepared at 300°C with 0.1M cobalt acetate, with 0.286 nm d-spacing belonging to the {220} family while Figure 11B shows a Co_3_O_4_ sample of semi-spherical morphology originating from non-faceted f-CoO, prepared at 321°C with 0.06M cobalt acetate, exhibiting {111} facets (Xie et al., 2009; Wen et al., 2014). High activity originating from Co_3_O_4_ [110] facets has been attributed to its enrichment with octahedrally coordinated Co^3+^ cations which have been shown to exhibit favorable CO adsorption properties and lower barriers for reaction between adsorbed CO and oxygen (Xie et al., 2009; Wang et al., 2012). To our knowledge, the finding that faceted h-CoO can be oxidized in air at high temperature to form faceted Co_3_O_4_ exposing the Co^3+^ enriched [110] surface is novel and presents a unique methodology for control over cobalt oxide surface structure.

## Conclusions

Iterative statistical design of experiments and high-throughput screening were used to study the effect of synthesis parameters in the one-pot colloidal synthesis of CoOx on nanoparticle properties and catalytic activity in CO oxidation. Of the six variables studied, it was found that the concentration of CoAc used in the synthesis together with the synthesis aging temperature had the most significant impact on nanoparticle structure and catalyst activity with light off temperatures below 90°C occurring above 0.06M CoAc and below 250°C. It was found that variations in light off temperatures with the CoAc concentration and aging temperature could largely be explained with the structure of the catalyst pre-calcination. In part, activity variations coincided with f-CoO particles with high degrees of grain boundary consolidation, η, formed at intermediate aging temperatures and CoAc concentrations which could enhance activity by providing active sites in close proximity to grain boundaries with fast oxygen diffusion from the bulk. Activity variations also coincided with the formation of h-CoO particles which exhibited preferential growth along the c-axis. The latter tended to form at low ramp rates, low aging temperatures, and high CoAc concentrations and were shown with HRTEM studies to oxidize into Co_3_O_4_ with highly active (110) surface facets and Co^3+^ enrichment. These findings demonstrate that Co^3+^ and grain boundary enrichment are the most significant structural variables in a design space assessing multiple properties including pre-calcination phase, morphology, and size, and provide a novel approach in the control of these properties.

## Author contributions

KM performed the experiments for this manuscript. JL and KM planned the experiments, interpreted the results, and write the manuscript.

### Conflict of interest statement

The authors declare that the research was conducted in the absence of any commercial or financial relationships that could be construed as a potential conflict of interest.
